# Common bile duct adenocarcinoma in a patient with situs inversus totalis: report of a rare case

**DOI:** 10.1186/1756-0500-5-681

**Published:** 2012-12-12

**Authors:** Hafida Benhammane, Saoussane Kharmoum, Sylvain Terraz, Thierry Berney, Thai Nguyen-Tang, Muriel Genevay, Omar El Mesbahi, Arnaud Roth

**Affiliations:** 1 Department of medical oncology, Hassan II University Hospital, Fez, Morocco; 2 Department of radiology, University Hospitals of Geneva, Geneva, Switzerland; 3 Department of general surgery, University Hospitals of Geneva, Geneva, Switzerland; 4 Department of gastroenterology, University Hospitals of Geneva, Geneva, Switzerland; 5 Department of pathology, University Hospitals of Geneva, Geneva, Switzerland; 6 Department of medical oncology, University Hospitals of Geneva, Geneva, Switzerland

**Keywords:** Situs inversus totalis, Bile duct cancer, Preoperative staging, Surgical management

## Abstract

**Background:**

Situs inversus totalis represents an unusual anomaly characterized by a mirror-image transposition of the abdominal and thoracic viscera. It often occurs concomitantly with other disorders that make difficult diagnosis and management of abdominal pathology. The relationship between situs inversus totalis and cancer remains unclear.

**Case presentation:**

We describe a 33-year old Guinean man with situs inversus totalis who presented with obstructive jaundice. Imaging and endoscopic modalities demonstrated a mass of distal common bile duct which biopsy identified an adenocarcinoma. The patient was successfully treated by cephalic pancreaticoduodenectomy followed by adjuvant chemoradiation and he is doing well without recurrence 8 months after surgery.

**Conclusion:**

The occurrence of bile duct adenocarcinoma in patient with situs inversus totalis accounts as a rare coincidence. In this setting, when the tumor is resectable, surgical management should be considered without contraindication and must be preceded by a careful preoperative staging.

## Introduction

Situs inversus totalis (SIT) is a rare congenital condition characterized by a mirror-image transposition of both the abdominal and thoracic viscera, its incidence accounts for 1/8000 to 1/25,000 of the normal population [1]. This condition may cause difficulties in the diagnosis and therapeutic management of abdominal pathology [2,3]. An increased risk of cardiac, splenic and hepatobiliary malformations are found in patients with SIT [4]; this abnormality is not considered to be a premalignant entity, however rare malignant neoplasms have been reported. A case of successfully treated common bile duct adenocarcinoma in a 33-year old man with SIT, an association described for the fourth time in the literature, is described.

## Case report

**Figure 1:**
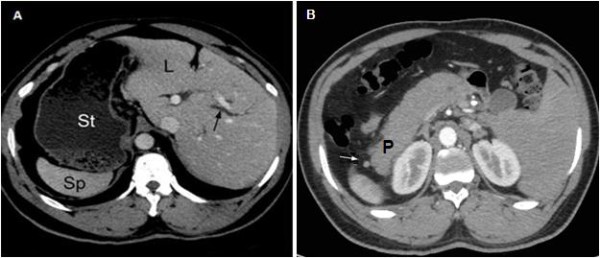
**A: Computed tomography during portal phase shows heterotopic location of the liver, the stomach and the spleen.** The intrahepatic bile ducts are mildly dilated (black arrow). **B**: There is also a small accessory spleen between the tail of the pancreas and the spleen (white arrow). L: liver; St: stomach; Sp: spleen; P: pancreas.

**Figure 2:**
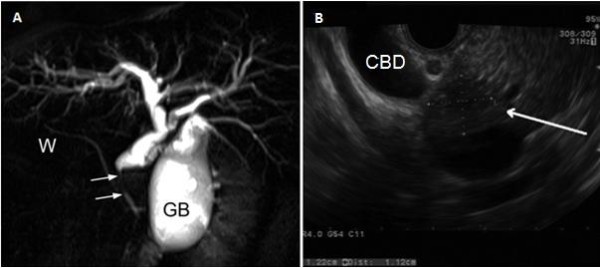
**A: Magnetic resonance cholangiopancreatography shows a tapered stricture of the common bile duct (arrows) with upstream dilation of the bile ducts.B**: Endoscopic ultrasonography shows an hypoechoic nodule (arrow) within the head of the pancreas and close to the dilated common bile duct. GB: gallbladder; W: Wirsung duct; CBD: common bile duct.

**Figure 3:**
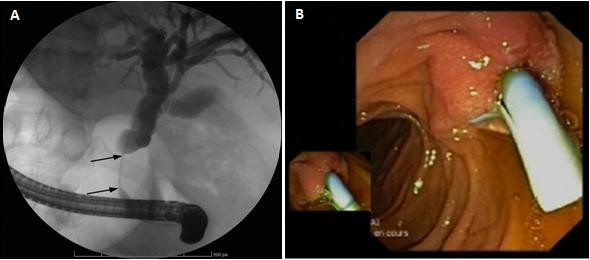
**A: Endoscopic retrograde cholangiopancreatography confirms the stricture of the common bile duct (arrows).B**: endoscopic view showing stenosis of distal common bile duct with placement of biliary stent.

**Figure 4:**
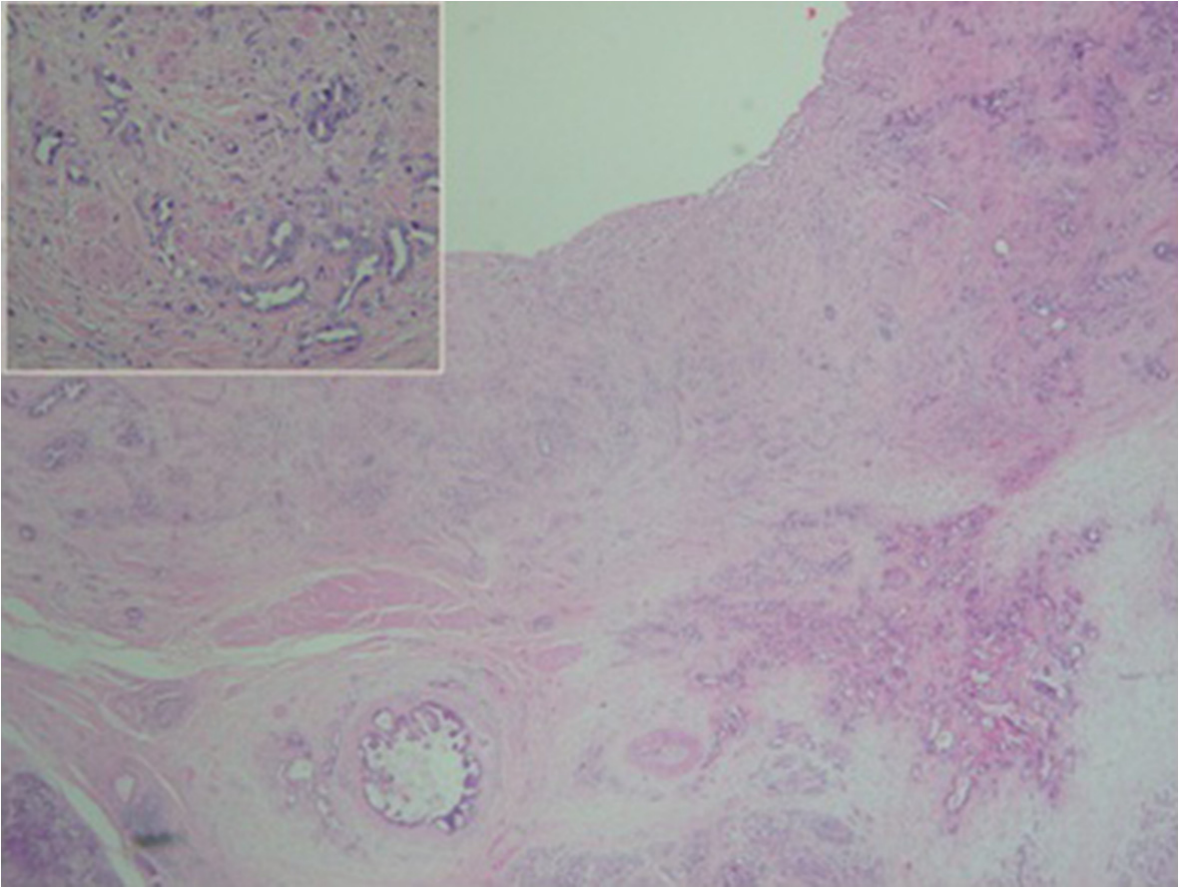
**Well differentiated adenocarcinoma centered by the common bile duct with very discrete infiltration of the surounding pancreatic parenchyma.** At the upper left of the image, morphology of the tumor at high magnification.

A 33-year old previously healthy man, with known SIT presented with 3 months history of obstructive jaundice and 10 Kg weight loss. Computed tomography (CT) confirmed the complete transposition of viscera as follows: dextrocardia, right subphrenic gastric bubble and spleen with a small accessory spleen, left sided liver and reversed superior vena cava, brachiocephalic artery and hepatic artery; CT also revealed a dilatation of intra and extra hepatic bile ducts secondary to stenosis of the distal common bile duct (CBD) (Figure 1). Magnetic resonance cholangiopancreatography confirmed the tapered stricture of the CBD with upstream dilation of the bile ducts (Figure 2A); Endoscopic ultrasound showed a 15 mm seized hypoechoic mass at the distal portion of the CBD (Figure 2B), which biopsies identified an adenocarcinoma with a strong positivity for Pancytokeratin. Staging showed no distant metastasis and serum levels of carbohydrate antigen 19-9 and carcinoembryonic antigen were normal. As the patient refused initially surgical management, an Endoscopic Retrograde Cholangio-Pancreatography (ERCP) with placement of biliary stent was performed (Figure 3); one year later the patient developed an iatrogenic acute pancreatitis and he finally agreed to the surgical approach. Following a left subcostal laparotomy, he underwent cephalic pancreaticoduodenectomy with resection of 17 lymph nodes and partial resection of portal and splenomesaraic vein, which were found to be invaded by the tumor. The procedure was evidently difficult and unusual because of the situs inversus. Histological analysis of the specimen resection confirmed the diagnosis of a well differentiated adenocarcinoma of CBD infiltrating the head of pancreas (Figure 4) with 5 metastatic lymph nodes in the retroportal lamina; biliary, pancreatic, duodenal and retroperitoneal margins were free of tumor, however the tumor was found to have invaded the venous section margin. The tumor was classified as pT3N1M0. The postoperative course was uneventful. Regarding the positive margin, complementary radiation with concurrent chemotherapy based on capecitabin was administrated. Currently, the patient is fit without any evidence of recurrence 8 months after surgical treatment.

## Discussion

Situs inversus is an uncommon congenital condition which is defined by a left-right transposition of the normally asymmetrical organs of the body as a mirror-image; transposition of thoracic and abdominal organs is termed situs inversus totalis [1]. This form occurs with a frequency of 1/ 8000 to 1/25000 and is characterized by dextrocardia with complete reversal of the heart chambers, the aorta turns to the right, the left lung has 3 lobes and the right lung only 2 [1,5]. In the abdomen, the stomach, spleen and pancreas are right sided of the body, the liver and gallbladder are left sided, the colonic flexures are reversed [4-6]. SIT occurs frequently with other disorders including Kartagener’s syndrome, primary ciliary dyskinesia, syndrome of asplenia or polysplenia as seen in our patient [1,4,7]. These anomalies are commonly associated with cardiovascular malformations which are more severe in the case of asplenia than in polysplenia [8]. The other anomalies include short pancreas, symmetric lobulation of the liver, biliary atresia, absence of the gallbladder and genitourinary anomalies; blood vessels, nerves and lymphatic are also transposed [1,7]. Pathogenic mechanisms of SIT have not been well elucidated. Some genetic patterns are involved including an autosomal recessive gene located to chromosome 14 [9] and deletions affecting chromosomes 7 or 8 [10]; Recently, significant advancements in understanding the possible molecular pathways were made suggesting that mutation affecting CCDC11 and DNAH11 genes are involved in autosomal recessive laterality defects of diverse phenotype resulting in SIT [11,12]. In addition, it has been shown that mutations in the TGF- β family gene and in the transcription factor HNF-3 β have a probable role in the process [13].

The association of SIT and neoplasia is a rare coincidence; only sporadic cases have been reported. The first case was published by Maekawa in 1927; it was an autopsy case of gastric carcinoma in a 43-year-old man with SIT [14]. SIT is not considered a premalignant condition; however, despite its rarity and regarding the non-negligible number of published sporadic cases of cancer in this setting, a relationship between situs abnormalities and cancer has been suggested. Some authors postulate that unidentified genes involved in left-right arrangement may have susceptibility of cancer like CCDC11 gene that mutation or loss expression is involved in some cancers [15,16]; in addition, it has been suggested that ciliary dysfunction via dysregulation of the hedgehog pathway may be the underlying cause of SIT [17], so Tomohiro et al. report that patients with SIT may be at high risk of developing cancer due to a congenital deficiency in the function of an intracellular protein, necessary to ciliogenesis, namely the KIF3 complex [18] leading to a ciliary dysfunction with a chronic inflammation which promote carcinogenesis via the pro-inflammatory nuclear factor kappa B [19]. These hypotheses remain not well elucidated and further research is needed to assess the genetic association between SIT and malignancy. Many different cancers have been reported (cancer of stomach, colon, pancreas, biliary tract, ampulla of Vater, and kidney). Biliary tract carcinomas are relatively rare, representing less than 1% of cancers; gallbladder adenocarcinoma and cholangiocarcinoma account for 4% and 3% of all gastrointestinal cancers respectively [20]. CT scan and ERCP are required for diagnosis and staging [21]. Complete surgical resection is the only potentially curative treatment available for resectable disease leading to 5-year survival rates of 5–10% for gallbladder cancer and 10–40% for cholangiocarcinoma [20,21]. In the setting of SIT, only three cases have been reported which were all cholangiocarcinoma [22-24]; herein we describe this association for the fourth time in literature; no case of gallbladder carcinoma has been reported. Careful preoperative staging by imaging modalities is important prior to invasive procedures in cancer patients with SIT in order to understand anatomic abnormalities [2,15]. Evidently, SIT makes the surgical procedure challenging because of the difficulties in following surgical protocols in this situation. However, the presence of anatomic variations should not modify the principles of oncologic surgery.

## Conclusion

SIT is an uncommon entity that often occurs concomitantly with other abnormalities. The relationship between SIT and cancer is not approved; further, studies are needed to identify precisely genetic and molecular patterns involved in development of malignancy in these patients. Because of the frequency of associated malformations of transposed organs and vascular and nervous anatomical variations that make difficult surgical management, special attention should be paid to the diagnosis and preoperative staging. Herein, we have described for the fourth time in the literature a case of unusual association of SIT and adenocarcinoma of common bile duct.

## Consent

Written informed consent was obtained from the patient for publication of this case report and any accompanying images. A copy of the written consent is available for review by the Editor-in-Chief of this journal.

## Abbreviations

SIT: Situs inversus totalis; CT: Computed tomography; ERCP: Endoscopic retrograde cholangiopancreatography; CBD: Common bile duct; CCDC11: Coiled-coil domain containing 11; DNHA1: Axonemal heavy chain dynein type 11; TGFβ: Tumors growth factor β; HNF-3β: Hepatocyte nuclear factor 3β; KIF3: Kinesin 3 family.

## Competing interests

The authors report no competing interests. The authors alone are responsible for the content and writing of the paper. All authors have contributed to this paper.

## Authors’ contributions

HB* was involved in the management of that patient, the analysis of the data and the literature research and wrote the manuscript. SK helped with the literature research. ST performed radiological diagnosis and revising manuscript. TB was involved in the surgical management of the patient. TNT was involved in the endoscopic diagnosis. MG performed histological diagnosis. OE helped with the final editing of the manuscript. AR approved the treatment and analyzed the literature data. All authors read and approved the manuscript.
